# Insights into the transmission of respiratory infectious diseases through empirical human contact networks

**DOI:** 10.1038/srep31484

**Published:** 2016-08-16

**Authors:** Chunlin Huang, Xingwu Liu, Shiwei Sun, Shuai Cheng Li, Minghua Deng, Guangxue He, Haicang Zhang, Chao Wang, Yang Zhou, Yanlin Zhao, Dongbo Bu

**Affiliations:** 1 Key Lab of Intelligent Information Processing, Institute of Computing Technology, Chinese Academy of Sciences, Beijing, China; 2 University of Chinese Academy of Sciences, Beijing, China; 3 City University of Hong Kong, China; 4 School of Mathematical Sciences, Center of Quantitative Biology, Center of Statistical Sciences, Peking University, Beijing, China; 5 Chinese Center for Disease Control and Prevention, Beijing, China; 6 State Key Laboratory of Theoretical Physics, Chinese Academy of Sciences, Beijing, China

## Abstract

In this study, we present representative human contact networks among Chinese college students. Unlike schools in the US, human contacts within Chinese colleges are extremely clustered, partly due to the highly organized lifestyle of Chinese college students. Simulations of influenza spreading across real contact networks are in good accordance with real influenza records; however, epidemic simulations across idealized scale-free or small-world networks show considerable overestimation of disease prevalence, thus challenging the widely-applied idealized human contact models in epidemiology. Furthermore, the special contact pattern within Chinese colleges results in disease spreading patterns distinct from those of the US schools. Remarkably, class cancelation, though simple, shows a mitigating power equal to quarantine/vaccination applied on ~25% of college students, which quantitatively explains its success in Chinese colleges during the SARS period. Our findings greatly facilitate reliable prediction of epidemic prevalence, and thus should help establishing effective strategies for respiratory infectious diseases control.

Respiratory infectious diseases pose a serious threat to human health, due to their high mortality rate as well as their rapid transmission across populations^1^. For example, influenza, a highly infectious disease, led to an estimated 250,000~500,000 deaths worldwide each year^2^. In comparison, tuberculosis, although spreading relatively slowly, was reported to cause more than 1.3 million deaths in 2012^3^. However, predicting the prevalence of respiratory infectious diseases and establishing effective control strategies remain fundamental challenges, partly due to the lack of understanding on the principles of disease spread.

Unraveling the principles of how respiratory infectious diseases spread requires a detailed understanding of human contact networks. Briefly, a human contact network consists of nodes representing individuals, as well as edges representing close proximity interactions (CPIs) among individuals^4^. Respiratory infectious diseases are predominantly transmit via air-borne droplets or aerosol among individuals with close proximity interactions; thus, the topological structure of CPI networks crucially affects transmission of respiratory diseases^5^.

Due to the lack of real CPI data, idealized models of human contact networks are commonly employed to study epidemic behavior. For instance, one such model, the homogeneous network, assumes any individual to interact with a fixed number of partners^1^,^5^. This assumption of uniform spread pattern was implicitly adopted by the classical differential-equation approach^6^. Another model is the heterogeneous network, such as small-world or scale-free network, which was introduced to capture the fact that in real life, some people are more socially connected than others^7^,^8^. However, these idealized models were not fully examined on the basis of real CPI data.

With the recent development of portable wireless technologies, such as Bluetooth, it is now possible to acquire real CPI data in an accurate and direct manner. For instance, two individuals wearing Bluetooth-enabled mobile phones can detect each other if the distance between them is within 10 meters, which also indicates a physically close proximity interaction between them. This CPI data acquisition approach is reasonable due to two aspects of the similarity between Bluetooth signal detection and respiratory disease spreading. First, airborne droplets, the major respiratory disease carrier, can travel for more than 6 meters (while coughing or sneezing)^9^, which is close to the detection range of Bluetooth signals. Second, both airborne droplets and Bluetooth signals are prone to be blocked by intermediate obstacles such as walls. Recently, several studies have been performed to probe real CPI patterns in US and French schools^10^,^11^,^12^ and residential communities^13^ using Bluetooth or similar wireless techniques.

CPI patterns have been reported to be highly related with lifestyle and demographics of human communities^10^. Thus, Chinese colleges might exhibit distinct CPI patterns, as these colleges exhibit a highly organized lifestyle of their student communities. Specifically, Chinese college students are organized into classes, and students within one class are also enrolled in identical courses. In addition, unlike schools in the US, most Chinese colleges are boarding schools, where undergraduate students live in neighboring dormitories on the same campus, and usually have their meals in large-capacity canteens.

To understand the disease spreading principles within Chinese colleges, we designed a mobile phone application called PEARL (Probing Entity Aggregation in Real Life) to collect CPIs through Bluetooth signal detection. Using this application, we acquired real CPI data among undergraduate volunteers in two representative Chinese colleges. Subsequently, we validated the acquired CPI networks by simulating the transmission of influenza and comparing the results with real influenza records. As applications of the real CPI networks, we investigated the disease spreading behavior in Chinese colleges. We also evaluated a variety of disease control strategies, with emphasis on class-cancelation strategy, which was widely applied in Chinese colleges during the SARS outbreak in 2003^14^.

## Methods

### Real CPI data collection

Using the PEARL application, we collected real CPI data amongst 174 undergraduates for 28 days (between October 31 and November 27, 2011) in a typical college in southern China (South China Agricultural University, or SCAU). To mitigate potential bias rooted at geographical locations, this data-gathering program was also carried out at a college in northern China (University of Science and Technology of Beijing, or USTB) to collect real CPI data from 87 undergraduates for 28 days (between October 24 and November 20, 2011). On the mobile phone of each volunteer, a PEARL client was installed to scan nearby PEARL clients via Bluetooth technique every 5 minutes. The detected Bluetooth addresses and scan time were recorded. Thus, in every 5 minutes, the CPI data was obtained among the individuals, from which a *basic network* was reconstructed with every node representing an individual, and every edge representing a 5-minute CPI among two individuals. The summation of all 288 (24 × 60/5 = 288) basic networks in a certain day formed a daily aggregation of CPI networks, where edge weights denote the aggregate CPI duration in the entire day. The PEARL CPI data sets are accessible via http://bioinfo.ict.ac.cn/pearl/.

Real influenza records were also collected from the SCAU undergraduate volunteers who participated in the PEARL program. In particular, all the volunteers were required to report their health condition from September 25 to October 21, 2011. Due to the memory loss, only 76 out of the total 174 volunteers reported this information. Among these volunteers, 13 were infected by influenza, but only 8 could remember the exact dates. Thus on a certain day, the number of volunteers who reported to be infected, denoted as #*infected*, can be calculated as


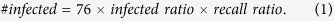


Here, *recall ratio* denotes the ratio of the infected volunteers who can recall the exact dates of infection. As mentioned above, recall ratio can be estimated as 8/13. Thus, the *infected ratio* was estimated as 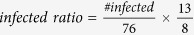 (Supplementary Table S1).

All data collection protocols were approved by Institute of Computing Technology, Chinese Academy of Sciences. Written informed consents were obtained from all participating volunteers. The methods were carried out in accordance with the approved guidelines.

### Simulating disease spread using SEIR model

The spreading process of respiratory infectious diseases was simulated using SEIR model^6^. SEIR model describes disease transmission processes where individuals transit step by step among four possible disease states, namely, susceptible (S), exposed/latent (E), infectious (I), and recovered (R).

At every step of epidemic simulation, every exposed individual becomes infectious with probability *δ*, while every infectious one causes each susceptible neighbor in the contact network (having interaction with the infectious one at that step) to be exposed with probability *β*, and infectious ones recover with probability *γ*.

The probabilities express how likely an individual changes its states at a step, and thus highly depend on how long a step takes (called unit time *t*). These probabilities were calculated as below.


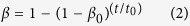



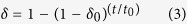



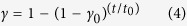


Here, the parameters *β*_0_, *δ*_0_, and *γ*_0_ denote respective probabilities within time *t*_0_.

Intuitively, 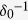 measures the duration of latent period, 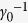 measures the duration of infectious period, and *β*_0_ measures how often a susceptible-infected contact results in a new exposure. The larger *β*_0_, the more rapidly diseases spread. Previous studies utilized different parameter settings for SEIR model. In ref. 15, 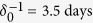, and 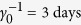; In ref. 16, 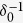 ranges over [1, 4] days, and 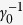 ranges over [3, 5] days; In ref. 17, 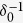 ranges over [1, 2] days, and 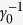 ranges over [1.5, 4] days; In ref. 4, *β*_0_ = 1.5 × 10^−4^ sec^−1^; In ref. 18, *β*_0_ was set as 1.5 × 10^−4^ sec^−1^ or 1.5 × 10^−4^ sec^−1^; In ref. 19, *β*_0_ was set as 2.8 × 10^−4^ sec^−1^ or 6.9 × 10^−4^ sec^−1^. Based on these studies, we set the parameter ranges as *β*_0_ in [0.1, 7.0] × 10^−4^ sec^−1^, 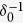 in [1, 4] days, and 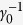 in [1.5, 5] days in this study.

The real CPI networks are usually constructed at a specific time resolution, as CPIs among individuals are dynamic. To simulate disease spread on dynamic CPI networks, the unit time of simulation was set according to time resolution. For example, the SCAU networks were constructed through Bluetooth scan every 5 minutes; thus, the unit time was set as 5 minutes accordingly for epidemic simulation. The unit time for other CPI networks used in this study are listed in Supplementary Table S2.

Our simulation starts with one of the individuals (called *index individual*) being exposed and the others being susceptible, and stops when no individuals are exposed or infectious. To remove the potential biases in index selection and state transition, a total of 10,000 simulations were performed with index individuals selected at random.

We considered the following four statistics of the simulation results: (1) The total infected percentage describes the percentage of individuals infected during the whole spreading process; (2) *R*_0_, also known as the basic reproductive number, represents the number of individuals infected directly by the index individual^6^. Epidemic outbreak occurs only when *R*_0_ > 1^5^. In this study, *R*_0_ is approximated as the average number of individuals infected by index individuals that are selected at random; (3) the peak epidemic time denotes when the number of infected individuals reaches its maximum value; and (4) the epidemic duration describes the duration of the disease spread.

### Evaluating disease control strategies

At present, the popular approaches to control the transmission of respiratory infectious diseases are targeted quarantine/vaccination, i.e., selecting a collection of individuals for quarantine or vaccination. The percentage of individuals selected is denoted as *quarantine/vaccination coverage*. A variety of network-based selection strategies have been proposed according to individual’s characteristics calculated based on CPI networks, including degree (contact number), strength, betweenness, clustering coefficient (CC), and the primary eigenvector^4^. In this study, the disease control strategies were simulated by removing the selected individuals from the CPI network followed by running the SEIR models. We evaluated the following network-based strategies for disease control, including degree strategy, strength strategy, CC strategy, betweenness centrality strategy, and eigenvector centrality strategy (Supplementary Methods).

Besides these network-based strategies, class cancelation is also commonly applied as a disease control strategy in boarding schools^14^. During class cancelation period in boarding schools, all students are ordered to stay within the campus, with all classes cancelled, thus showing a CPI pattern nearly identical to that at weekends. In our study, class cancelation was simulated by simply replacing the CPI networks at weekdays with the CPI networks at weekends.

The platform for disease spread simulating and quarantine/vaccination strategies evaluating are accessible via http://bioinfo.ict.ac.cn/pearl/.

## Results

### Analyzing real CPI networks in two representative Chinese colleges

**Figure 1:**
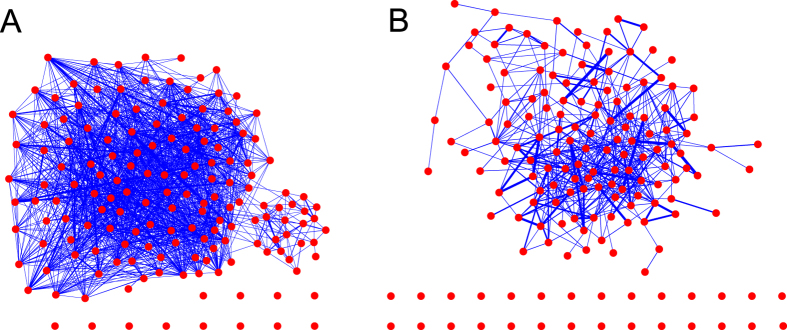
Real CPI networks acquired on October 31 (Monday) and November 5 (Saturday), 2011 in SCAU, denoted as (**A**) SCAU1031 and (**B**) SCAU1105, respectively. Here, a node represents an individual student. If there is a CPI between two students, an edge is drawn between the two corresponding nodes with line width proportional to the aggregate CPI duration in the entire day. The two CPI networks are shown as examples since the CPI networks on weekdays exhibit nearly identical characteristics, and so do those on weekends (Supplementary Fig. S1).

Using the PEARL application, we collected real CPI data from two representative Chinese colleges, namely, SCAU, and USTB. Figure 1 shows two examples of the SCAU CPI networks acquired on October 31, 2011 (weekday, denoted as SCAU1031) and November 5, 2011 (weekend, denoted as SCAU1105), where nodes represent students and edges represent CPIs, with line width proportional to the aggregate interaction duration during one day. Supplementary Fig. S2 shows two examples of USTB CPI networks in the same manner.

In fact, the real CPIs can be divided into two categories according to interaction duration, namely, transient CPIs formed by purely random interactions, and regular CPIs representing meaningful interactions among students^4^. We therefore employed a mixture statistical model to describe the CPI durations (Supplementary Methods S1), and determined the optimal duration threshold to distinguish these two categories as 30 minutes (Supplementary Fig. S3). To remove the biases in network statistics introduced by purely random CPIs, the CPIs with an aggregate duration of less than 30 minutes were filtered out before calculating network statistics.

To understand the detailed characteristics of real CPI networks, a collection of idealized CPI networks was constructed for comparison, including small-world (SW) networks generated using the Newman-Watts model, scale-free (SF) networks generated using the Barabási-Albért model, and uniformly-random (UR) networks generated using the Erdös-Rényi model^20^. For unbiased comparison, all the idealized CPI networks were generated with identical numbers of individuals and CPIs to counterpart real CPI networks (Supplementary Methods). To understand the distinct characteristics of CPI networks in Chinese colleges, we compared them with the real CPI networks acquired using Bluetooth and similar wireless techniques from a US undergraduate dormitory (denoted as USD)^11^, a US high school (denoted as USHS)^4^, and a French primary school (denoted as FRPS)^12^. For both real and idealized CPI networks, a variety of descriptive statistics were calculated, including degree distribution, CC, efficiency, modularity, and periodicity (see Supplementary Methods for detailed definitions).

It is worth pointed out that the generated idealized networks are un-weighted, and the network statistic calculations are applicable only to un-weighted networks, too. For fair comparison, un-weighted version of real CPI networks should be employed. A straightforward strategy to achieve this objective is to simply ignore the duration of real CPIs, no matter how strong or weak they are. However, this strategy might lead to deviation in calculation of network statistics, as CPIs greatly vary in durations (Figs 1 and S2). In fact, the CPI durations can be perfectly fitted by the mixture of a Poisson distribution and a truncated Gaussian distribution (Supplementary Methods and Supplementary Fig. S3). These two distributions were used to describe transient CPIs formed by purely random interactions, and regular CPIs representing meaningful interactions among students, respectively. Based on the intersection point of these two distributions, a threshold to separate transient and regular CPIs can be reasonably determined as 30 minutes, i.e., CPIs with duration no more than 30 minutes were treated as transient. Here, only regular CPIs were considered in calculations of network statistics, and idealized networks were generated with same size to the real CPI networks with transient CPIs removed.

The analysis of network statistics are described as below.

**Figure 2:**
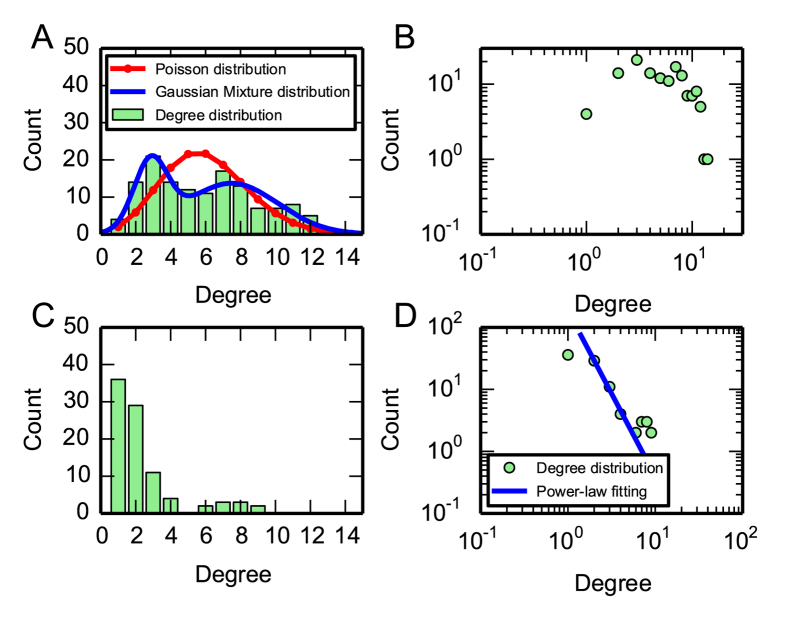
Degree distribution of SCAU1031 network (panels A,B) and SCAU1105 network (panels C,D). Panels A and C are shown in linear scale, while panels B and D are shown in log-log scale. As shown in panel A, the degree distribution of the SCAU1031 network can be well fitted by applying a mixture of two Gaussian distributions (blue line), which significantly deviates from the idealized Poisson distribution of the counterpart uniformly-random network with the same number of nodes and edges (red line). As shown in panel B, the SCAU1031 network exhibits a degree distribution significantly different from the power-law distribution. Thus, the CPI data acquired on weekdays are neither uniformly random nor scale-free. In contrast, SCAU1105, a CPI network acquired on a weekend, exhibits a typical power-law degree distribution (panels C,D).

**Figure 3:**
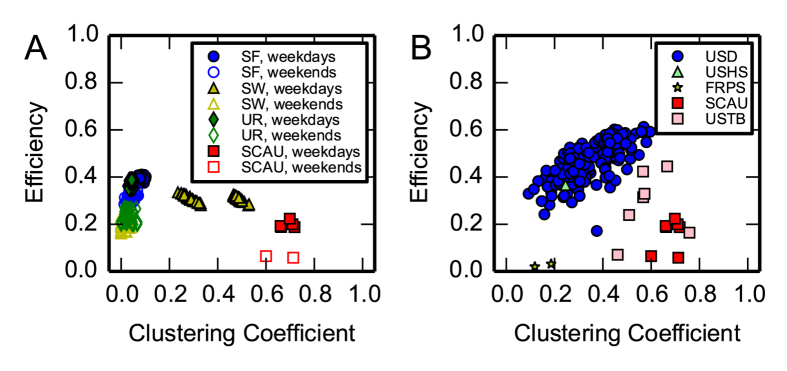
Clustering coefficient and efficiency of real and idealized CPI networks. (**A**) SCAU CPI networks and the counterpart scale-free (SF), small-world (SW), uniformly random (UR) networks of identical size. The SCAU CPI networks on weekdays are substantially different from the idealized CPI networks, and so do the networks on weekends. (**B**) Real CPI networks acquired from SCAU, USTB, the US and French schools. The SCAU and USTB CPI networks are different from the CPI networks acquired from the schools in the US and France. Here, only results from one typical week of SCAU networks (between October 31 and November 6, 2011) and USTB networks are shown.

- **Degree distribution**: In a CPI network, the degree of an individual refers to the number of partners that have interactions with this individual. It is well known that a uniformly random network approximates a Poisson degree distribution, and that a scale-free network is featured by its power-law degree distribution; thus, degree distribution is commonly used as a criterion to judge whether a network is scale-free or uniformly random^20^.As shown in Fig. 2, the SCAU1031 network has a degree distribution that is significantly different from both the Poisson distribution and power-law distribution. Thus, the CPI networks acquired on weekdays are neither uniformly random nor scale-free. However, SCAU1105, a CPI network acquired on a weekend, exhibits a typical power-law degree distribution. This observation is consistent with the viewpoint that on weekends, CPI networks are mainly determined by friendship among students^10^, and friendship networks usually exhibit a power-law degree distribution^21^. Similar observations were made from other real CPI networks in SCAU (shown in Supplementary Fig. S4).
- **CC and efficiency**: These two features are commonly used to quantify the small-world behavior of networks. Specifically, CC describes the extent to which nodes in a network tend to cluster, and efficiency measures the speed at which bacteria or viruses spread across a human contact network. In general, a small-world network has a high CC but low efficiency; thus, CC and efficiency are commonly used as criteria to judge whether a network is small-world or not^20^. As shown in Fig. 3A, our SCAU networks on weekdays from October 31 to November 4, 2011 have a CC of ~0.70 while the CC of the counterpart small-world networks has a maximum of 0.55. Furthermore, the CC of SCAU networks on the weekends (November 5 and 6, 2011) are at least 0.60, which are substantially higher than that of the counterpart small-world networks (less than 0.1). However, the efficiency of SCAU networks were found to be lower than that of the corresponding small-world networks, especially those on weekends. Together, these statistics demonstrated that SCAU networks are not small-world networks. Our SCAU networks also exhibited higher CC than that in the US and French schools. The SCAU CPI networks displayed a lower efficiency than that studied in the US schools, but higher than that observed in the French school. Interestingly, the USTB networks exhibited a similar pattern to that of SCAU networks except for the larger deviations of CC as well as efficiency.
- **Modularity**: As shown in Supplementary Fig. S5, the network SCAU1031 consists of a set of tightly interacting modules. This observation was confirmed by quantitative analysis of modularity, which measures the degree that a network can be separated into disconnected communities. There, the SCAU networks possess an average modularity of ~0.82, which is considerably higher than that of the counterpart small-world (0.62), scale-free (0.23), and uniformly-random networks (0.25). Supplementary Fig. S5 also reveals the complicated dynamics of communities, including community merging and diversification. Therefore, it is not sufficient to understand CPI patterns merely based on the CPI network acquired on a single day. In addition to the comparison with idealized networks, SCAU networks were also compared with real CPI networks acquired in the US and French schools. As shown in Table 1, the SCAU CPI networks exhibit an average modularity (0.78) that is significantly higher than USD (0.49) and USHS (0.43) networks.
- **Periodicity**: A strong periodicity of CPI networks was expected according to the weekly school schedule of college students. However, similarity analysis of the daily CPI networks from October 31 to November 27, 2011 shows that this periodicity is weak (Supplementary Fig. S6), highlighting the necessity of investigating long-term real CPI data.

In summary, our analysis provided us with two main insights into the statistics of real CPI networks. First, the CPI networks in the two representative Chinese colleges are different from the CPI networks acquired from the US as well as French schools. One explanation is that Chinese college students commonly have highly organized lifestyle, such as living in neighboring dormitories on the same campus, having meals in large-capacity canteens altogether, and being organized into classes for taking identical courses. This highly organized lifestyle is reflected in the formation of a set of small but tightly-interacting communities. Second, the SCAU CPI networks acquired on weekdays are neither scale-free, small-world, nor uniformly random. This challenges the basic assumption of idealized CPI networks adopted in traditional approaches to epidemic research^5^,^6^,^7^,^8^. Such assumption might result in a large deviation in disease dynamics, which has been confirmed by the difference in epidemic dynamics across real CPI networks with that across idealized CPI networks (shown below).

### Simulating influenza spread across real CPI networks

Using a SEIR model, we simulated the spread of influenza across the acquired CPI networks, and compared with real influenza records^4^,^6^. Unlike the calculation of network statistics, all CPIs, including both transient and regular ones, were considered in epidemic simulation. In fact, simply ignoring transient CPIs would lead to deviations in epidemic behavior (Supplementary Fig. S7).

**Figure 4:**
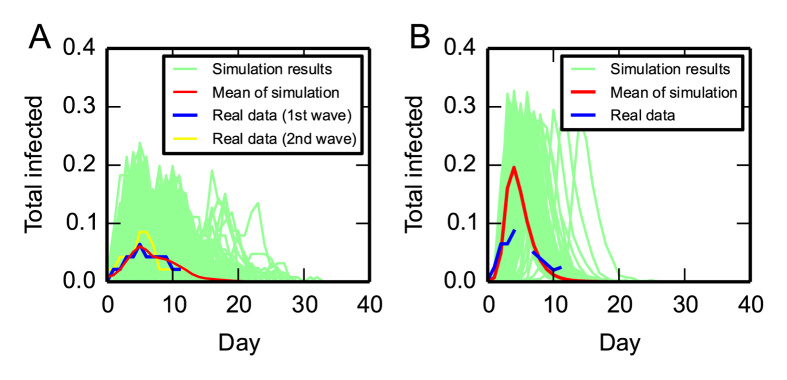
Simulation results of respiratory infectious disease spreading across (**A**) SCAU CPI networks and (**B**) USHS CPI networks correspond to the records of real influenza outbreaks. The *x*-axis represents days from the first infection event, and the *y*-axis represents the percentage of the infected population. We used the same setting of SEIR parameters for simulations in both SCAU and USHS (*β*_0_ = 2.5 × 10^−4^ sec^−1^, 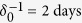, 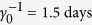). In SCAU, the influenza epidemic occurred in two waves: the first wave (green) occurred between September 29 and October 9, and the second wave (blue) occurred between October 9 and October 21, 2011. The two waves were aligned to put the disease onset date at the point of origin. In USHS, the influenza records were derived from absentee data^4^. As shown in panels A and B, the simulation results are in good accordance with the real influenza records in both SCAU and USHS. In panel B, the incomplete blue line corresponds to a gap in the original absentee data^4^.

Among the widely-used ranges of SEIR parameters^4^,^15^,^16^,^17^,^18^,^19^, the parameter setting that best fit the real SCAU infection data was determined as *β*_0_ = 2.5 × 10^−4^ sec^−1^, 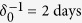, 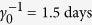. As shown in Fig. 4, the disease prevalence prediction using SCAU CPI networks is in good accordance with real influenza records of the SCAU undergraduate volunteers. Using the same setting of SEIR model parameters, we repeated the disease spread simulation across the USHS CPI networks^4^. Similarly, we observed that the disease prevalence prediction using USHS CPI networks is in good accordance with the real influenza records in USHS. These results justified both parameter setting and epidemic simulation for studying epidemic dynamics of respiratory infectious diseases.

**Figure 5:**
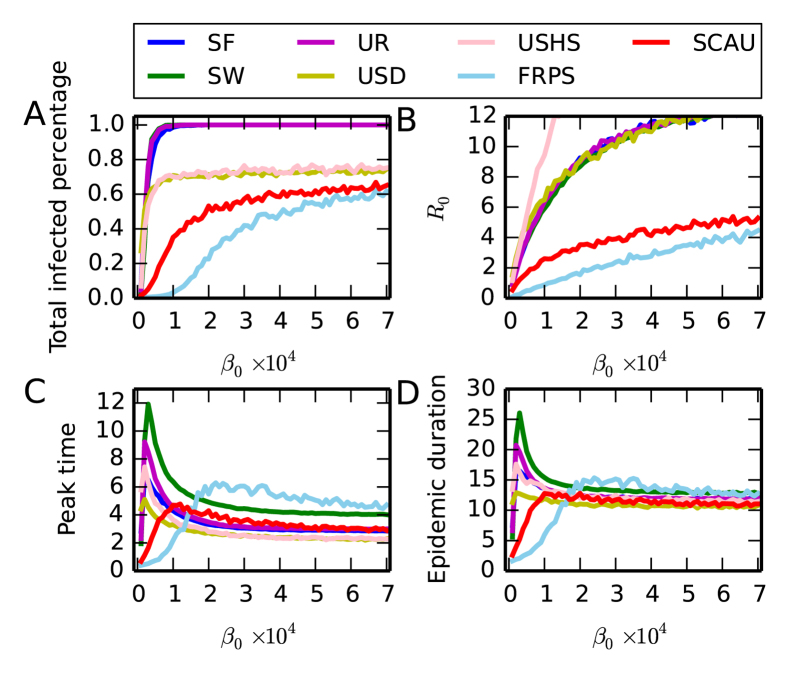
Comparison of SEIR simulations over real CPI networks (including USD, USHS, FRPS, and SCAU CPI networks), and idealized CPI networks (including scale-free, small-world, and uniformly random networks). The comparisons include terms of (**A**) total infected population ratio, (**B**) *R*_0_, i.e. the expected number of cases infected directly from the index case, (**C**) peak time, i.e. the time when the number of infected cases reached its maximum, and (**D**) epidemic duration, i.e., the duration from the introduction of the index case until the recovery of the last case. SEIR parameters: 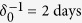, 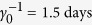, and *β*_0_ ranges from 0.1 to 7.0 × 10^−4^ sec^−1^.

To investigate whether idealized CPI networks can also be used to study disease transmission, we compared real CPI network from different regions as well as idealized CPI networks in term of epidemic behavior. Surprisingly, idealized CPI networks substantially differ from the real SCAU CPI networks in simulating spreading of influenza as well as infectious diseases that transmit relatively slowly (Fig. 5). Specifically, in the simulation of influenza spreading using idealized small-world networks, almost all individuals were found to be infected, and the *basic reproductive number R*_0_ was estimated to be as high as 9.5. In contrast, the percentage of individuals infected is only 55%, and *R*_0_ is only 3.8 when real SCAU CPI networks were used for simulation (Fig. 5A,B). Furthermore, the epidemic dynamics of infectious diseases that transmit relatively slowly was simulated by setting relatively small parameter *β*_0_ ≤ 1.0 × 10^−4^ sec^−1^. As shown in Fig. 5A, all individuals are infected in the simulations using idealized CPI networks; however, the percentage of infected individuals is less than 35% in the simulations using real SCAU CPI networks. In addition, the disease spreading across real SCAU CPI networks shows early peak epidemic time but short epidemic duration (Fig. 5C,D). These results reveal the potential risk of overestimating disease prevalence when simply using scale-free, small-world, or uniformly random networks in epidemic simulations.

Furthermore, in order to understand the effects of CPI patterns on influenza spreading behavior, we repeated the epidemic simulation using the CPI networks acquired from the US and French schools^4^,^11^,^12^, and compared the simulation results with that in Chinese colleges. Figure 5 clearly shows the differences of these schools in disease spreading behavior. First, in both the simulations of influenza and the simulations of the diseases that transmit relatively slowly, the percentages of total individuals infected in SCAU and FRPS are less than half of those of the US schools. Second, the simulations across SCAU and FRPS networks show a *R*_0_ of less than 3, which is substantially smaller than that in USD and USHS CPI networks (USD: 6.5, USHS: 9.4). This small *R*_0_ implies a relatively slow transmission in SCAU and FRPS. Third, for the diseases that transmit relatively slowly, a delayed peak epidemic time is observed in US schools compared to those in SCAU and FRPS. Similar observations were also obtained when the SCAU networks are replaced with the USTB networks, implying that these observations are general to Chinese colleges (Supplementary Fig. S8).

**Figure 6:**
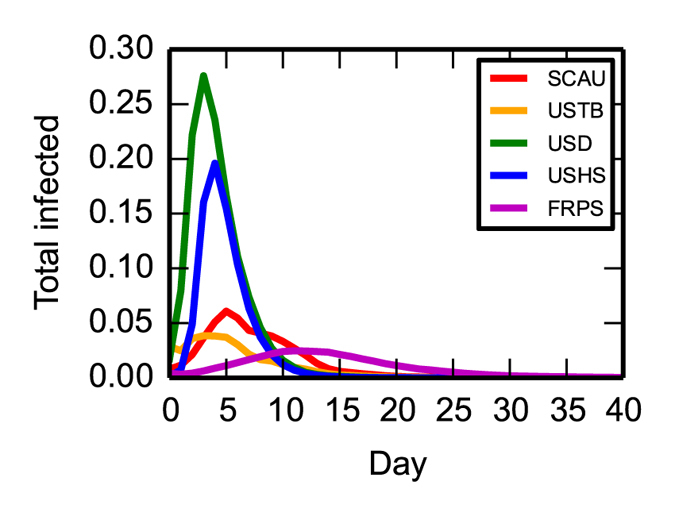
Percentage of the infected population reported by SEIR simulations over real CPI networks in USD, USHS, FRPS, SCAU, and USTB. Here, the *x*-axis denotes the time starting from the index individual being set as *infected*, and *y*-axis denotes the ratio of infected individuals. Here the SEIR parameters *β*_0_ = 2.5 × 10^−4^ sec^−1^, 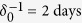, 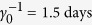 were used to simulate influenza spreading.

In fact, the influenza spreading behavior can be clearly divided into two categories: (1) in the US schools, disease outbreak appears to be relatively strong (peak prevalence: 0.20) and early (peak time: ~2 days); (2) in the schools of SCAU, USTB as well as FRPS, a considerably low transmission (peak prevalence: 0.06) was observed (Fig. 6).

It is worth pointed out that epidemic simulation of respiratory infectious diseases requires a sequence of daily CPI networks, as disease transmission usually lasts for several days. Due to the lack of long-term CPI data, previous studies were required to construct a sequence of CPI networks whereby a CPI network acquired on a particular day was simply repeated. In contrast, we gathered 28-day real CPI data in two representative Chinese colleges, allowing an examination of the previously-used network construction strategies. Our experimental results reveal that the previously-used strategy of network construction results in a substantial deviation from the 28-day CPI real networks in epidemic simulation (Supplementary Fig. S9). One underlying reason for this observation is related to the fact that by simply repeating a single day’s CPI network, the community dynamics, such as merging, vanishing or expansion of network communities during the evolving process of long-term CPI networks, cannot be captured (Supplementary Fig. S5). Therefore, a CPI network acquired on any single day may not be representative of the entire CPI network sequence. Our study also reveals that the CPI network data obtained in seven consecutive days yielded simulation results close to that of the entire 28-day CPI networks, indicating the CPI data obtained for seven days can be used for epidemic research of disease spreading in schools (Supplementary Fig. S9).

Together, the real CPI networks acquired in the two Chinese colleges show different epidemic dynamics from both idealized networks and real CPI networks acquired in the US schools. This difference might be attributed to the strong modularity of undergraduates in Chinese colleges, since disease spreading is usually limited within communities. Due to this difference, it cannot be assumed that the nature of CPI networks is scale-free, small-world, or uniformly random networks in epidemic analysis; furthermore, using CPI networks acquired from a certain region as a universal contact model should be avoided when studying epidemic behavior.

### Applying the real CPI networks to evaluate disease control strategies

**Figure 7:**
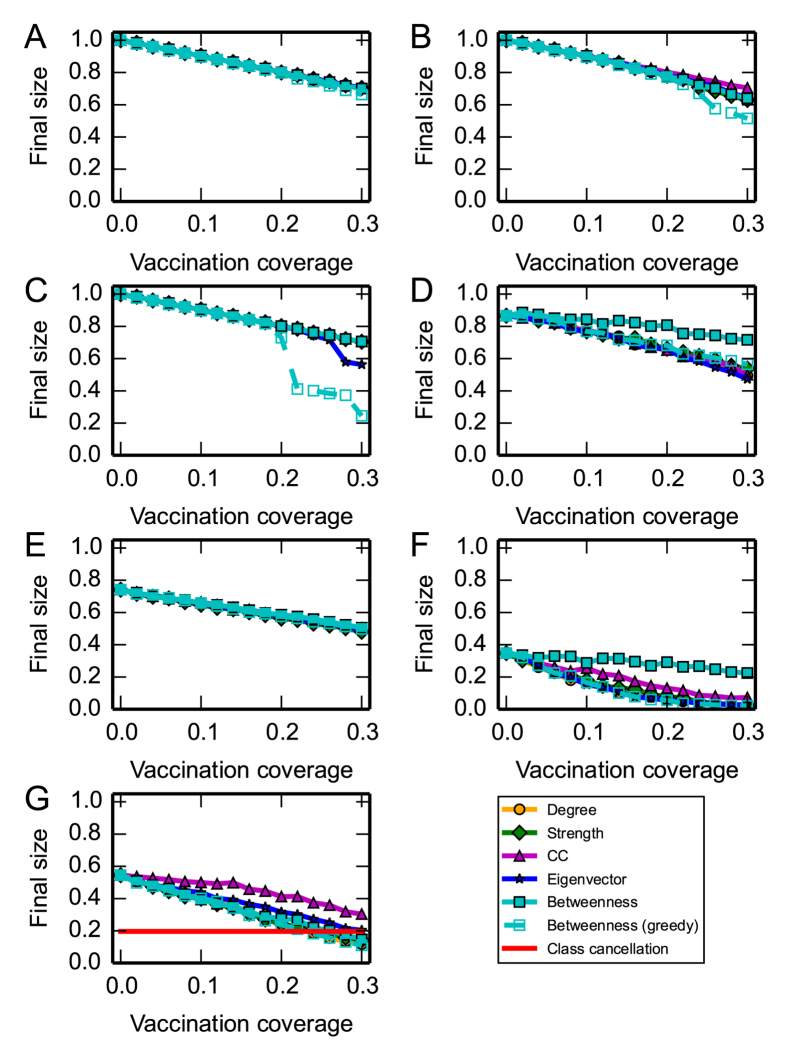
Mitigating power of various quarantine/vaccination strategies over (**A**) uniformly random, (**B**) scale-free, (**C**) small-world, (**D**) USD, (**E**) USHS, (**F**) FRPS, and (**G**) SCAU CPI networks. Here, the *x*-axis denotes vaccination coverage, and the *y*-axis denotes the total population infected. As quarantine/vaccination coverage increases, the total number of individuals infected drops in a linear pattern (the slopes are −1.0, −1.3, −0.9, and −1.2 for uniformly random, scale-free, small-world, and SCAU CPI networks, respectively). In addition, the targeted vaccination strategies were found to be extremely effective in SCAU and FRPS than in the US schools. Remarkably, class cancelation, though simple, is ranked as the most effective strategy for disease control as it shows a mitigating power equal to quarantine/vaccination applied on ~25% of college students. Here the SEIR parameters *β*_0_ = 2.5 × 10^−4^ sec^−1^, 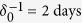 and 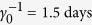 were used to simulate influenza spreading.

The success of applying real CPI networks in epidemic simulations allowed for an evaluation of various strategies for disease control. The class cancellation strategy, as well as a total of five network-based strategies (degree, strength, betweenness, CC, and the primary eigenvector), were evaluated, and the evaluation results are described as below. As shown in Fig. 7, most network-based strategies exhibit nearly identical power in mitigating disease spread. Specifically, as quarantine/vaccination coverage increases, the total number of individuals infected generally decreases in a linear pattern. In order to avoid the biases rooted in parameter setting, a second setting of SEIR model parameter *β*_0_ = 1.0 × 10^−4^ sec^−1^, 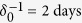, 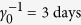 was also evaluated, with similar observations obtained (Supplementary Fig. S10).

Figure 7D–G suggest that the effectiveness of targeted quarantine/vaccination strategies is CPI-dependent. Specifically, the targeted quarantine/vaccination strategies are extremely effective in SCAU and FRPS relative to that in the US schools. For example, by applying the *betweenness centrality* selection strategy on the SCAU CPI networks, the ratio of population infected drops from 0.52 to 0.39 under the quarantine/vaccination coverage of 10%, and further drops to 0.27 under the quarantine/vaccination coverage of 20%. On FRPS networks, the ratio of population infected is observed to be as small as 0.05 under a quarantine/vaccination coverage of 20%. In contrast, the ratio of population infected remains higher than 0.50, even at a quarantine/vaccination coverage as high as 30% in both USD and USHS.

Remarkably, the class-cancelation strategy shows a significant mitigating power of reducing the ratio of population infected by approximately 70% (Fig. 7G). This mitigating power is better than all the five network-based quarantine/vaccination strategies even when the quarantine/vaccination coverage is as high as 25%. Class cancelation was one of the most frequently used control strategies against the outbreak of SARS in China in 2003^14^; however, the power of class cancellation has never been thoroughly evaluated. The simulation presented here provides quantitative supports for the success of this strategy.

## Discussion

Here we present long-term real CPI data acquired from two representative Chinese colleges, and compare them with the real CPI networks acquired from the schools in US and France. These real CPI networks, together with epidemic simulations, showed the distinct transmission pattern of respiratory infectious diseases in Chinese colleges, and quantitatively characterized mitigating power of the class cancelation strategy. Specifically, our findings can be summarized as below:

- Real CPI networks have different characteristics from idealized CPI networks. In addition, the real CPI networks acquired from Chinese colleges are significantly different from those acquired from the schools in the US and France, which is possibly caused by the highly organized lifestyle of Chinese college students.
- Simulated epidemic spread on real CPI networks is different from that on idealized networks, usually generating less infection cases. Real CPI networks from different settings, such as schools in China and the US, also showed different epidemic behavior.
- Class cancelation, though simple, is considered as an effective quarantine strategy for Chinese colleges.

In addition, realistic long-term CPI networks cannot be accurately constructed by simply repeating 1-day’s CPI data. On the contrary, repeating 7-day’s CPI data is probably adequate, which is consistent with the weekly school schedule.

Apart from these new insights into the CPI pattern within Chinese colleges, there are several limitations of our study to be taken into consideration. First, the CPI data was gathered from 174 volunteers, which covered only ~15% of the entire undergraduate community in the Information School of SCAU. On one hand, we demonstrated, theoretically and experimentally, that the network characteristics CC and degree density are insensitive to the coverage of CPI data gathering (Supplementary Methods, and Fig. S11). In addition, epidemic simulations across the acquired CPI networks are in good agreement with real influenza outbreak records (Fig. 4). On the other hand, such small coverage might affect the calculation of some network statistics^22^, and a large-scale data collection program would help clarifying the extent to which the coverage of CPI data gathering affects estimation of network statistics.

Another limitation lies at the low recall rate of infections and additional uncertainty of infection dates. In our study, only 76 out of 174 volunteers reported their health condition, reaching a recall rate of 44%. Another dataset of CPI networks along with epidemic records would facilitate our understanding of epidemic behavior across CPI networks.

In addition, our study was built on the hypothesis that air-borne droplets and Bluetooth signals share comparable transmission distances. It is assumed that Bluetooth signal travels up to ~10 meters^23^; in contrast, the transmission distance of air-borne droplets is based on estimation only, with no exact data available at present^9^. A detailed investigation, together with improved measuring techniques, will help clarifying the transmission distance of air-borne droplets in the future.

Together, our study presents real CPI data in representative Chinese colleges, and reports how the special CPI pattern affects respiratory disease transmission and disease control. Our findings should greatly facilitate the prediction of epidemic prevalence in colleges as well as other human communities, and thus allow for designing more effective disease control strategies than those available at present.

## Additional Information

**How to cite this article**: Huang, C. *et al*. Insights into the transmission of respiratory infectious diseases through empirical human contact networks. *Sci. Rep.* **6**, 31484; doi: 10.1038/srep31484 (2016).

## Supplementary Material

Supplementary Information

## Figures and Tables

**Table 1:** Real CPI networks collected from a US undergraduate dormitory (USD), a US high school (USHS), a French primary school (FRPS), a college in northern China (USTB), and a college in southern China (SCAU).

CPI Network	Sources	Population size	Duration	Modularity
USD	An undergraduate dormitory in the US^11^	70	2008.10–2009.5	0.491 (±0.068)
USHS	A high school in the US^4^	788	a typical school day	0.431
FRPS	A primary school in France^12^	242	2 days in October, 2009	0.676 (±0.002)
USTB	A college in northern China	87	28 days (2011)	0.623 (±0.081)
SCAU	A college in southern China	174	28 days (2011)	0.784 (±0.031)
