# Supplementary material for: Insights into the transmission of respiratory infectious diseases through empirical human contact networks
Source: Sci Rep. 2016 Aug 16;6:31484. doi: 10.1038/srep31484 (PMC4985757; doi:10.1038/srep31484)
Supplement: Supplementary Information [file srep31484-s1.pdf]

Insights into the transmission of respiratory infectious diseases through empirical human contact networks
(Supplementary Information)

Chunlin Huang, Xingwu Liu, Shiwei Sun,
Shuai Cheng Li, Minghua Deng, Guangxue He,
Haicang Zhang, Chao Wang, Yang Zhou,
Yanlin Zhao & Dongbo Bu

June 6, 2016

1 Methods

1.1 Distinguishing regular CPIs from transient CPIs formed by purely random interactions

As shown in Fig. 2 in the paper, the duration of interactions vary greatly. In fact, the real CPIs can be divided into two categories according to CPI duration, namely, the transient CPIs formed by purely random human interactions, and regular CPIs representing meaningful interactions among students [1]. Thus, we employed a mixture statistical model to describe the CPI durations, and determined the optimal duration threshold to distinguish these two categories.

As shown in Supplementary Fig. S3, the CPI duration histogram of the SCAU network on November 18, 2011 shows two peaks, where the left peak (denoting transient CPIs) can be approximated by a geometric distribution, and the right one (denoting regular CPIs) can be approximated by a Poisson distribution.

Consider N CPIs with durations denoted as $x_1, \cdots, x_N$. Suppose the parameters of the geometric distribution and Poisson distribution are p and λ respectively. Then the distribution of CPI duration can be described by a mixture model as below.

$$Pr(x_n = k) = \pi_1 (1-p)^{k-1} p + \pi_2 \frac{\lambda^k e^{-\lambda}}{k!}. \tag{1}$$

Here, π_1, π_2 denote the weight of the two distributions, respectively. The parameters π_1, π_2, p, λ were calculated using the expectation-maximization (EM)

technique, where the expectation step (E-step) and maximization step (M-step) were alternatively executed until convergence.

E-step: At the $(t+1)$-st iteration, for each CPI duration sample x_n ($n = 1, 2, ...N$), we calculated the likelihoods for x_n being a transient CPI or regular CPI using the parameters $\pi_1^{(t)}, \pi_2^{(t)}, p^{(t)}, \lambda^{(t)}$ estimated at the t-th iteration. In particular, we have:

$$L_1(x_n) = \frac{\pi_1^{(t)}(1-p^{(t)})^{x_n-1}p^{(t)}}{\pi_1^{(t)}(1-p^{(t)})^{x_n-1}p^{(t)} + \pi_2^{(t)}\frac{(\lambda^{(t)})^{x_n}e^{-\lambda^{(t)}}}{x_n!}} \tag{2}$$

$$L_2(x_n) = \frac{\pi_2^{(t)}\frac{(\lambda^{(t)})^{x_n}e^{-\lambda^{(t)}}}{x_n!}}{\pi_1^{(t)}(1-p^{(t)})^{x_n-1}p^{(t)} + \pi_2^{(t)}\frac{(\lambda^{(t)})^{x_n}e^{-\lambda^{(t)}}}{x_n!}} \tag{3}$$

and

$$N_1 = \sum_{n=1}^{N} L_1(x_n), N_2 = \sum_{n=1}^{N} L_2(x_n) \tag{4}$$

M-step: Update parameter estimations $\pi_1^{(t+1)}, \pi_2^{(t+1)}, p^{(t+1)}, \lambda^{(t+1)}$ as below:

$$p^{(t+1)} = \frac{N_1}{\sum_{n=1}^{N} L_1(x_n)x_n}, \tag{5}$$

$$\lambda^{(t+1)} = \frac{1}{N_2}\sum_{n=1}^{N} L_2(x_n)x_n \tag{6}$$

and

$$\pi_1^{(t+1)} = \frac{N_1}{N}, \pi_2^{(t+1)} = \frac{N_2}{N} \tag{7}$$

Having determined the two distributions, the intersection point can be used as the distinguishing threshold for transient interactions. In the example, the distinguishing threshold is calculated as 6 Bluetooth scans, which equals to 30 mins since the Bluetooth scan is repeated every 5 minutes (Supplementary Fig. S3). Similar observations were obtained from CPI networks on other dates due to the stability of transient CPI distribution.

1.2 Construction of idealized CPI networks

Idealized networks were constructed from real CPI networks. Two different scenarios existed here: (1) constructing one idealized network from one real CPI network, which was utilized in network analysis; and (2) constructing one idealized network from several consecutive real CPI networks, which was used in epidemic simulation.

1. Constructing one idealized network from one real CPI network Suppose the real network has n individuals, and m CPIs with weights $w_1, \cdots, w_m$. Then the idealized network to be constructed should have n individuals, and m CPIs with identical weight $\hat{w} = \frac{1}{m}\sum_i w_i$.

2. Constructing one idealized network from several consecutive real CPI networks Suppose there are T real networks, which have identical number of n individuals but different number of CPIs (m_t CPIs for the t-th network) with different weight ($w_{t,i}$ for i-th CPI in t-th network). Then, the idealized network will have n individuals and $\hat{m}$ CPIs with identical weight $\hat{w}$, where $\hat{m} = \frac{1}{T}\sum_t m_t$ and $\hat{w} = \frac{1}{Tm}\sum_{t,i} w_{t,i}$.

With determined number of individuals and CPIs with identical weight, we constructed three different idealized network: uniformly-random network, scale-free network, and small-world network. The construction process was based on Erdös-Rényi model, Barabási-Albért model, and Newman-Watts model, respectively [2]. Suppose the real CPI network has n individuals and m CPIs with identical weight w. The algorithms for the construction are as follows, where we use "nodes" to denote "individuals", and use "edges" to denote "CPIs'.

1.2.1 Generate uniformly-random networks

Uniformly-random networks are generated simply from n isolated nodes, and randomly connecting two nodes until m edges are formed (Algorithm 1).

Algorithm 1 Construct uniformly-random network

```
1: Construct a network with n isolated nodes.
2: while number of edges < m do
3:     Randomly select two unconnected nodes, and connect them with an edge
       weighted w.
4: end while
```

1.2.2 Generate scale-free networks

In scale-free network generating process, we first initialize n_0 isolated nodes, and at every step later, we add a new node and connect it to Δm old nodes with w-weighted edges. The first newly added node should be connected to *all* of the n_0 old nodes to avoid isolated nodes in the final network. Thus, if we connect every new node to n_0 old ones, we have

$$\frac{m}{n - n_0} = n_0,$$

and we get $n_0 = \frac{n-\sqrt{n^2-4m}}{2}$. To make sure n_0 is an integer, we set

$$n_0 = \lfloor \frac{n - \sqrt{n^2 - 4m}}{2} \rfloor. \tag{8}$$

Since we have connected n_0 edges in the network when the first new node is added, we set the number of new edges in the following every step as

$$\Delta m = \frac{m - n_0}{n - n_0 - 1}. \tag{9}$$

If Δm is not an integer, we apply a small trick to achieve our goal of m total edges, by adding $\lceil \Delta m \rceil$ edges with probability of $\Delta m - \lfloor \Delta m \rfloor$, and adding $\lfloor \Delta m \rfloor$ edges with probability of $\lceil \Delta m \rceil - \Delta m$. The construction process is shown in Algorithm 2.

Algorithm 2 Construct scale-free network

```
Calculate the number of initial isolated nodes: n_0 = ⌊(n − √(n^2 − 4m))/2⌋;
Calculate the number of edges to add in each step of BA model: Δm = (m − n_0)/(n − n_0 − 1);
Construct a network with n_0 isolated nodes.
Add a node and connect it to previously isolated n_0 nodes with weight w.
while graph size < n do
  Add a new node;
  if Δm is not an integer then
    Calculate
```

$$\begin{aligned} \Delta m_1 &= \lceil \Delta m \rceil \\ \Delta m_2 &= \lfloor \Delta m \rfloor \\ p_1 &= \Delta m - \Delta m_2 \\ p_2 &= \Delta m_1 - \Delta m \end{aligned}$$

```
    Let Δm' = Δm_1 with probability p_1, or Δm = Δm_2 with probability p_2;
  else
    Let Δm' = Δm;
  end if
  Select Δm' nodes randomly with probability proportional to their degrees,
  and connect them to the newly added node with weight w.
end while
```

1.2.3 Generate small-world networks

In small-world network generating process, we first initialize a regular circle with n nodes and m_0 edges, and then randomly add m_1 edges. The number of edges used in the initial circle must be a multiple of n, thus $m_0 = n\lfloor \frac{m}{n} \rfloor$, and $m_1 = m - m_0$. The detailed construction is shown in Algorithm 3.

1.3 Periodicity analysis of CPI networks

Taking the school schedule into considerations, one might expect strong periodicity from CPI networks in schools. To investigate periodicity of our CPI networks, we calculated the similarity between two aggregation CPI networks N_i and $N_{i+\Delta t}$, where N_i denote the CPI network at i-th day. If there is a periodicity of Δt, we will observe considerable similarities between N_i and $N_{i+\Delta t}$

Algorithm 3 Construct small-world network

Calculate the number of initial edges in the circle: $m_0 = n\lfloor \frac{m}{n} \rfloor$.
Calculate the initial degree $d_0 = \frac{2m_0}{n}$.
Generate a circular network with each node connecting to d_0 neighbors, and all edge weights are w.
while number of edges $< m$ **do**
    Randomly choose two unconnected nodes, and connect them with an edge weighted w.
end while
for every node **do**
    for every edge connected to the node **do**
        Rewire the edge towards another unconnected and randomly chosen node with probability of 0.01.
    end for
end for

for all i. The similarities were measured using the following 4 features.

- Degree distribution similarity:

$$\mathrm{Corr}_{\mathrm{degree}}(\Delta t) = \frac{1}{T-\Delta t}\sum_{j=1}^{T-\Delta t}\frac{\sum_{k=1}^{n}(d_k^i-\bar{d}^i)(d_k^j-\bar{d}^j)}{\sqrt{\sum_{k=1}^{n}(d_k^i-\bar{d}^i)^2\sum_{k=1}^{n}(d_k^j-\bar{d}^j)^2}} \quad (10)$$

  where i is defined as $i = j+\Delta t$, d_k^i denotes the degree of the k-th individual in N_i, and $\bar{d}^i$ denotes the average degree of N_i.

- Strength distribution similarity:

$$\mathrm{Corr}_{\mathrm{strength}}(\Delta t) = \frac{1}{T-\Delta t}\sum_{j=1}^{T-\Delta t}\frac{\sum_{k=1}^{n}(s_k^i-\bar{s}^i)(s_k^j-\bar{s}^j)}{\sqrt{\sum_{k=1}^{n}(s_k^i-\bar{s}^i)^2\sum_{k=1}^{n}(s_k^j-\bar{s}^j)^2}} \quad (11)$$

  where i is defined as $i = j + \Delta t$, s_k^i denotes the strength of the k-th individual in N_i, and $\bar{s}^i$ denotes the average strength in N_i.

- Average fraction of common neighbors:

$$F_{\mathrm{cn}}(\Delta t) = \frac{1}{T-\Delta t}\sum_{j=1}^{T-\Delta t}\frac{1}{n}\sum_{k=1}^{n}\frac{CN(k,N_i,N_j)}{d_k^j} \quad (12)$$

  where i is defined as $i = j+\Delta t$, d_k^i denotes the degree of the k-th individual in N_i, $CN(k,N_i,N_j)$ denotes the number of common neighbors of the k-th individual shared by N_i and N_j.

- Average fraction of repeated contacts:

$$F_{\mathrm{rc}}(\Delta t) = \frac{1}{T-\Delta t}\sum_{j=1}^{T-\Delta t}\frac{1}{n}\sum_{k=1}^{n}\frac{RC(k,N_i,N_j)}{s_k^j} \quad (13)$$

where i is defined as $i = j + \Delta t$, s_k^i denotes the strength of the k-th individual in N_i, s_{kl}^i denotes the number of contacts between the k-th and the l-th individuals on the i-th day, and $RC(k, N_i, N_j) = \sum_{l=1}^{n} \min\{s_{kl}^i, s_{kl}^j\}$ denotes the number of repeated contacts of the k-th individual in N_i and N_j.

For the consecutive 28 SCAU aggregation CPI networks, these similarity features were calculated and shown in Supplementary Fig. S6. The figure demonstrates a periodicity of 7 days, which is consistent with the school schedule. However, the periodicity is not so strong; thus, the long-term CPI networks cannot be constructed by simply repeating one week of CPI networks.

1.4 The relationship between CPI data characteristics and data-collection coverage

The CPI data in SCAU were gathered from 174 volunteers, which cover only ∼10% of the whole undergraduate community. The small sample size might affect the calculation of some network properties [3], and a large-scale data collection program in the future would help clarify the extent to which the sample size affects the network properties estimation and understanding of the disease spread. However, this is not an inherent drawback as we can demonstrated, theoretically and experimentally, that the network characteristics of CC, strength distribution are insensitive to the sample size. In other words, the sample size in the study is sufficient for most of the network analysis (Supplementary Fig. S10).

Suppose that the underlying CPI network (denoted as $\mathcal{A}$) among the whole community has N nodes and M edges, and the acquired CPI network (denoted as $\mathcal{B}$, which is a random sample of $\mathcal{A}$) has n nodes and m edges. We will prove that the density and CC of the sampled network $\mathcal{B}$ are good approximations to those of the underlying network $\mathcal{A}$.

1.4.1 The stability of density

Theorem 1. *Suppose $\mathcal{B}$ is a random sample of $\mathcal{A}$. The expectation of the density of $\mathcal{B}$ equals to the density of $\mathcal{A}$.*

Proof. Let denote the N nodes of $\mathcal{A}$ as $A_1, A_2, \cdots, A_N$, and

$$a_{ij} = \begin{cases} 1 & \text{if } A_i \text{ and } A_j \text{ have a contact} \\ 0 & \text{otherwise} \end{cases} \quad \text{where } i = 1, 2, \cdots, N; j = 1, 2, \cdots, N.$$

B_i $(i = 1, 2, \cdots, n)$ and b_{ij} $(i = 1, 2, \cdots, n, j = 1, 2, \cdots, n)$ are defined similarly.

Consider the sampling process as a permutation π, where a subset C of n nodes are first randomly selected from the N nodes in $\mathcal{A}$, and are then permuted

randomly. Then we have

$$2m = \sum_{p=1}^{n}\sum_{q=1}^{n} b_{pq} \tag{14}$$

$$= \sum_{i\in C, j\in C, i\neq j} b_{\pi(i)\pi(j)} \tag{15}$$

Note that $b_{\pi(i)\pi(j)}$ is binary and the probability $\Pr(b_{\pi(i)\pi(j)} = 1) = \frac{M}{\frac{1}{2}N(N-1)}$ since the nodes in $\mathcal{B}$ are randomly selected from the nodes in $\mathcal{A}$. Thus we have

$$E(m) = \frac{1}{2}\sum_{i\in C, j\in C, i\neq j} E(b_{\pi(i)\pi(j)}) \tag{16}$$

$$= \frac{1}{2}n(n-1)\frac{M}{\frac{1}{2}N(N-1)} \tag{17}$$

$$= \frac{n(n-1)}{N(N-1)}M \tag{18}$$

Therefore, the expectation of the density of $\mathcal{B}$ equals to the density of $\mathcal{A}$.

□

1.4.2 The stability of clustering coefficient

Theorem 2. *Suppose $\mathcal{B}$ is a random sample of $\mathcal{A}$. The expectation of clustering coefficient of $\mathcal{B}$ equals to the clustering coefficient of $\mathcal{A}$.*

Proof. There are two different definitions of clustering coefficients (CC). Here, we prove the theorem under two definitions, respectively.

1. The clustering coefficient of a network is defined as the mean CC of all nodes. Consider a specific node v with degree D in $\mathcal{A}$. Suppose there are a total of M edges among the D nodes. Then the CC of v is defined as:

   $$CC_A(v) = \frac{M}{\frac{1}{2}D(D-1)} \tag{19}$$

   Suppose in the network $\mathcal{B}$, the node v has a degree of d and there are a total of m edges among the d nodes. Then the CC of v in $\mathcal{B}$ can be calculated as:

   $$CC_B(v) = \frac{m}{\frac{1}{2}d(d-1)} \tag{20}$$

   Thus, we have $E(CC_B(v)) = CC_A(v)$ since $E(m) = \frac{d(d-1)}{D(D-1)}M$.

2. Another definition is that CC can be calculated as three times the ratio of the number of triangles and triples.

   Suppose in the sampling process to generate $\mathcal{B}$ from $\mathcal{A}$, every node is selected with the probability of p. Thus, every connected triple, as well as every triangle, is selected with the probability of p^3. Hence, we have $E(CC_B(v)) = CC_A(v)$.

□

1.5 The network-based strategies for disease control

At present, the popular approaches to control the transmission of respiratory infectious diseases are targeted quarantine/vaccination, i.e., selecting a collection of individuals for quarantine or vaccination. The percentage of selected individuals is denoted as *quarantine/vaccination coverage*. A variety of network-based selection strategies have been proposed according to individual's characteristics calculated based on CPI networks, including degree (contact number), strength, betweenness, clustering coefficient (CC), and the primary eigenvector.

In this study, we evaluated the following network-based strategies for disease control.

1. Degree strategy: The quarantine/vaccination subjects were selected according to the number of CPI partners, i.e., the node degree in CPI networks. The individuals were ranked with descending order of degrees, and were removed in this order until the required vaccination coverage was achieved. The rationale underlying this selection is to perform quarantine/vaccination on the individuals with frequent CPIs with others.

2. Strength strategy: The quarantine/vaccination subjects were selected according to the total duration of interaction with others. The ranking and removing policy is identical to that used in the degree strategy.

3. CC strategy: For an individual, CC denotes the fraction of the number of its CPI partner pairs that have interactions over the total possible number of its CPI partner pairs. A high CC usually means that the individual is unlikely to constitute a bottleneck for disease spread; thus, we chose nodes with lower CC for quarantine/vaccination, leading to the opposite policy. The removing policy is identical to that used in the degree strategy.

4. Betweenness centrality strategy: An individual has high betweenness centrality if it lies on shortest paths of many individual pairs. Specifically, for an individual i, its betweenness centrality is defined as $C_B(i) = \sum_{s \neq t \neq i} \frac{\sigma_{st}(i)}{\sigma_{st}}$, where s and t denotes two individuals in the network, σ_{st} denotes the total number of shortest paths between s and t, and $\sigma_{st}(i)$ is the number of those paths passing by i. All individuals ranked in nonincreasing order of betweenness centralities, and the top k individuals were selected for quarantine/vaccination such that the required coverage was achieved.

5. Betweenness centrality strategy (greedy version): After the individual with the highest betweenness centrality was removed, the betweenness centrality of every remaining individual was updated, and the next candidate for removing was selected accordingly. This process was repeated until the required coverage was achieved.

6. Eigenvector centrality strategy: Eigenvector centrality of a vertex is the corresponding component of the primary eigenvector of the adjacency matrix A, where entry a_{ij} denotes the CPIs duration between individuals i and j. Intuitively, an individual with high eigenvector centrality is more likely to be a hub of the CPI network. The ranking and removing policy is identical to that used in the degree strategy.

Besides these network-based strategies, class cancelation is also commonly applied as a disease control strategy in boarding schools. During class cancelation period in boarding schools, all students are ordered to stay within the campus, with all classes cancelled, thus showing a CPI pattern nearly identical to that at weekends. In the study, class cancelation was simulated by simply replacing the CPI networks at weekdays with the CPI networks at weekends.

2 SI Tables

Table S1: **Real influenza records collected from the SCAU undergraduate volunteers who participated in the PEARL program.**

Date	#Infected	Infected ratio
09-29	1	0.0214
09-30	1	0.0214
10-01	2	0.0428
10-02	2	0.0428
10-03	3	0.0641
10-04	2	0.0428
10-05	2	0.0428
10-06	2	0.0428
10-07	2	0.0428
10-08	1	0.0214
10-09	1	0.0214
10-10	1	0.0214
10-11	2	0.0428
10-12	2	0.0428
10-13	2	0.0428
10-14	4	0.0855
10-15	4	0.0855
10-16	3	0.0641
10-17	1	0.0214
10-18	1	0.0214
10-19	1	0.0214
10-20	1	0.0214
10-21	1	0.0214

Table S2: Unit time used for SEIR simulations

Network	Unit time
SCAU	5 minutes
USTB	5 minutes
USHS	20 seconds
USD	5 minutes
FRPS	20 seconds
Scale-free	24 hours
Small-world	24 hours
Uniformly random	24 hours

3 SI Figures

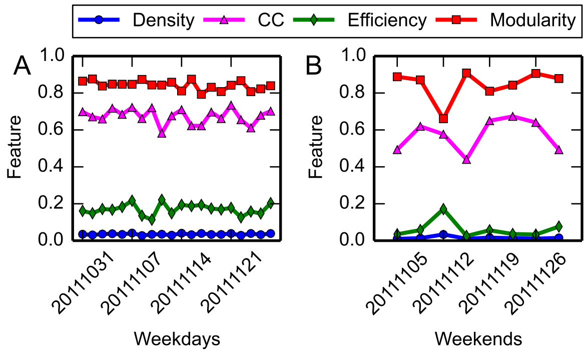

Figure S1: The characteristics of SCAU CPI networks on weekdays (A) and weekends (B). The characteristics include density (blue), CC (magenta), efficiency (green) and modularity (red). It can be observed that all CPI networks on weekdays show similar characteristics, and so do the CPI networks on weekends. The only exception is the CPI network on November 12th — there was an activity in SCAU, which results in relatively frequent transient interactions. These frequent transient interactions further lead to significantly low modularity.

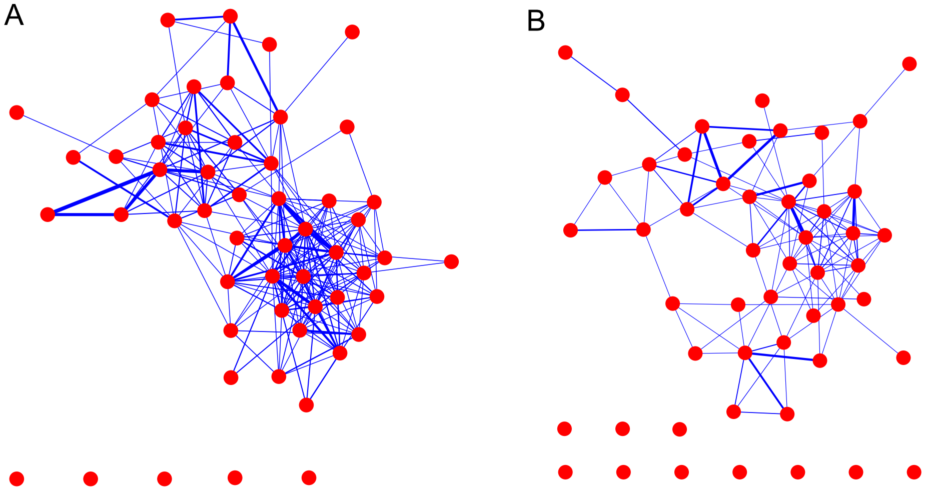

Figure S2: Real CPI networks acquired on November 07 (Monday) and November 12 (Saturday), 2011 in USTB, denoted as (A) USTB1107 and (B) USTB1112, respectively. Here, a node represents an individual student, and if there is a CPI between two students, an edge is drawn between the two corresponding nodes with edge width proportional to the aggregate CPI duration in the entire day.

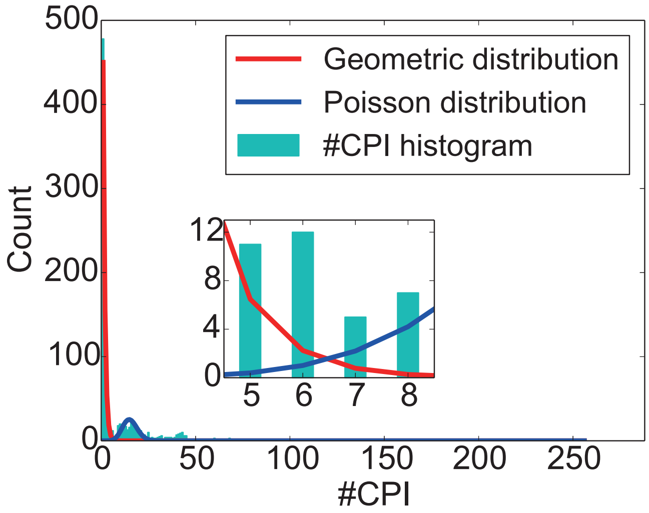

Figure S3: Histograms of CPI durations among SCAU volunteers on November 18, 2011. The histogram is approximated with the mixture of a geometric distribution and a Poisson distribution, plotted with red and blue lines, respectively. The inset shows in details two lines interacting at CPI = 6.5 scans, which implies that the contacts lasting less than or equal to 6 bluetooth scan (or $6 \times 5 = 30$ minutes) are transient interactions.

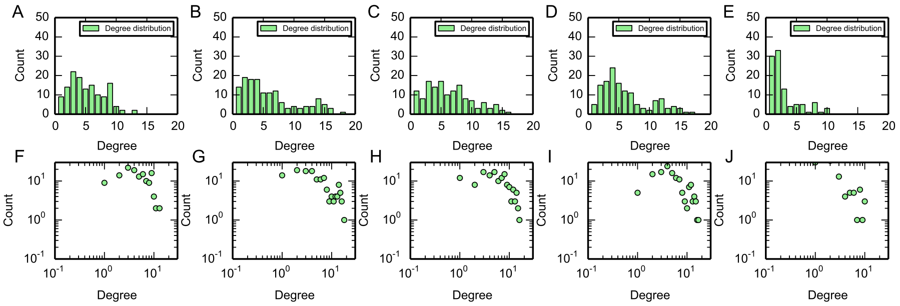

Figure S4: Degree distribution of real CPI networks acquired on November 1 (Tuesday, panels A and F), November 2 (Wednesday, panels B and G), November 3 (Thursday, panels C and H), November 4 (Friday, panels D and I), and November 6 (Sunday, panels E and I), 2011 in SCAU, denoted as SCAU1101, SCAU1102, SCAU1103, SCAU1104 and SCAU1106 respectively. Panels A-E are shown in linear scale, while panels F-J are shown in log-log scale. It can be observed that the CPI networks on weekdays (SCAU1101, SCAU1102, SCAU1103, SCAU1104) significantly deviate from idealized Poisson distribution of the counterpart uniformly-random network with the same number of nodes and edges. In contrast, the network on the weekend (SCAU1106) shows a power-law distribution.

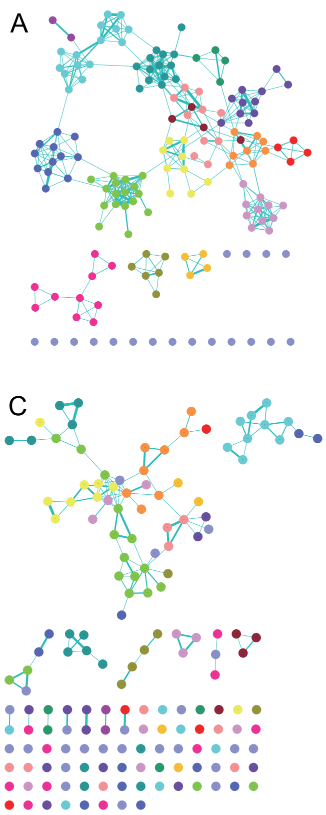

Figure S5: Community structures of SCAU CPI networks on a consecutive 4 days, namely, (A) October 31 (Monday), (B) November 1 (Tuesday), (C) November 5 (Saturday) and (D) November 7 (Monday), 2011. The edge widths shown are proportional to the corresponding CPI duration. The communities of SCAU1031 network were identifed and drawn in different colors. In the other 3 networks, the individuals are drawn in the same color used in SCAU1031; thus, the community dynamics—including merging, vanishing, and creation—can be clearly observed.

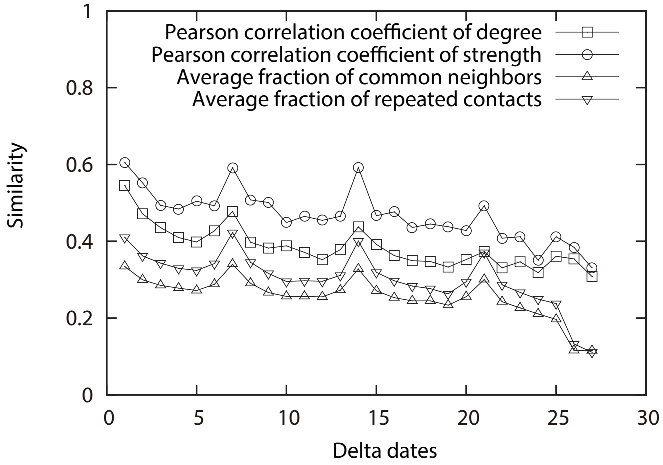

Figure S6: The function of the similarity between two daily aggregation CPI networks on the time difference. The similarity measures include Pearson CC of degree, Pearson CC of strength, average fraction of common neighbors, and the ratio of common contacts. The statistics suggest a periodicity of about seven days, which is consistent with the school schedule. However, the similarities are not as strong as expected.

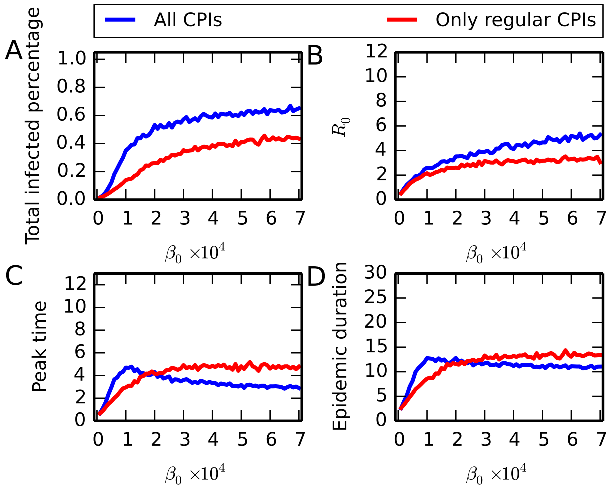

Figure S7: SEIR simulation results over SCAU networks with transient CPIs (blue line) and without transient CPIs (red line). A collection of simulation results are shown here, including (A) total infected population ratio; (B) R_0; (c) peak time; and (d) epidemic duration. The removal of transient CPIs leads to significant deviation of epidemic behavior. SEIR parameters: $\delta_0^{-1} = 2$ days, $\gamma_0^{-1} = 3$ days, and β_0 ranges from 0.1 to 7×10^{-4} sec^{-1}.

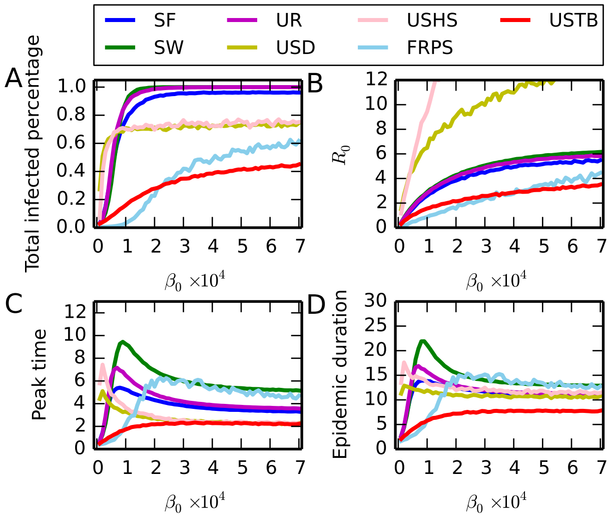

Figure S8: Comparison of SEIR simulations over real CPI networks (including USD, USHS, FRPS, and USTB CPI networks), and artificial CPI networks (including scale-free, small-world, and uniformly random networks). The artificial CPI networks were constructed with the identical number of nodes and edges to USTB networks. The figure shows a collection of simulation results, including (A) total infected population ratio; (B) R_0, i.e., the expected number of cases infected directly from the index case; (C) peak time, i.e. the time of maximum infected cases and (D) epidemic duration, or the period of time from the introduction of the index case until recovery from the infection of the last case has occurred. SEIR parameters: $\delta_0^{-1} = 2$days, $\gamma_0^{-1} = 1.5$days, and β_0 ranges from 0.1 to 7.0×10^{-4}. For each parameter setting, the SEIR simulations were repeated 10,000 times with calculated mean values.

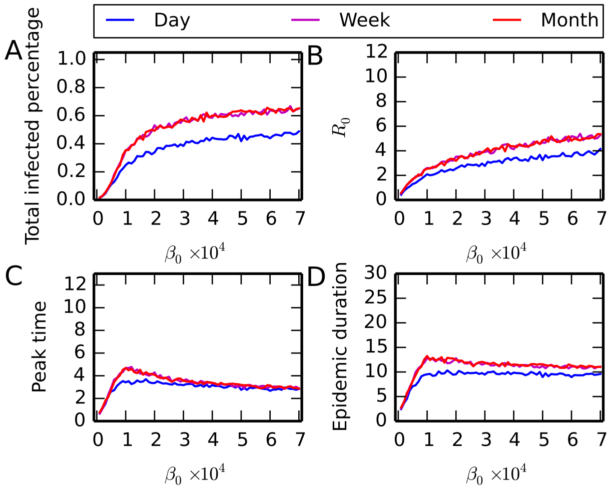

Figure S9: SEIR simulation results over a sequence of CPI networks, which were constructed via different strategies, including (*i*) repetition of a certain day's network (SCAU1118); (*ii*) repetition of one week's CPI networks (SCAU1031-1106); and (*iii*) the original 28 days' CPI networks. A collection of simulation results were shown here, including (A) total infected population ratio; (B) R_0, i.e., the expected number of cases infected directly from the index case; (C) peak time, i.e., the time of maximum infected cases and (D) epidemic duration, or the time period from the introduction of the index case until the recovery of the last case. SEIR parameters: $\delta_0^{-1} = 2$ days, $\gamma_0^{-1} = 1.5$ days, and β_0 ranges from 0.1 to 7.0×10^{-4}. For each parameter setting, the SEIR simulations were repeated 10,000 times with calculated mean values.

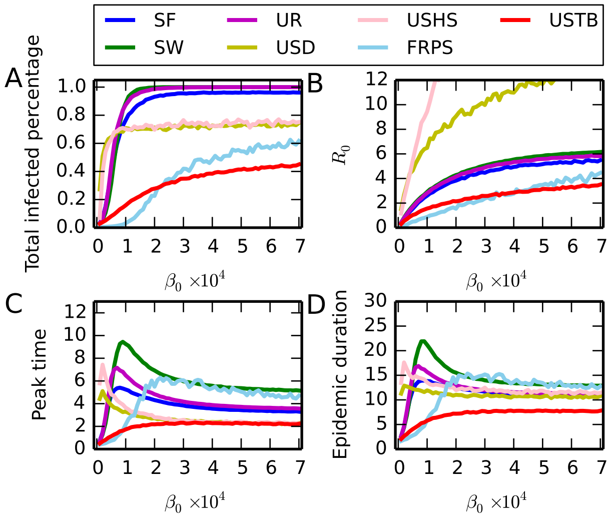

Figure S10: Mitigating power of various quarantine/vaccination strategies over (a) UR, (b) SF, (c) SW, and (d) SCAU CPI networks. Here, the x-axis denotes vaccination coverage, and the y-axis denotes the total number of the infected population. SEIR model parameters: $\beta_0 = 1.0 \times 10^{-4}$ sec^{-1}, $\delta_0^{-1} = 2$ days and $\gamma_0^{-1} = 3$ days.

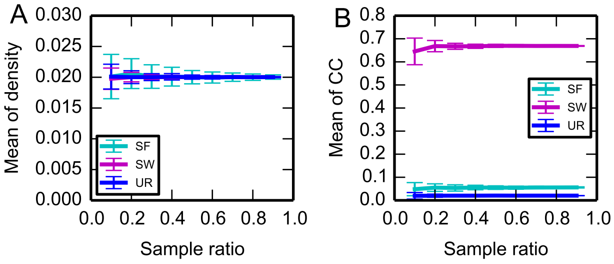

Figure S11: The function of mean values of network characteristics on the coverage. The network characteristics include (A) density, and (B) CC. At each coverage level, a total of 100 random subsets of vertices were selected and 100 subgraphs were induced from these vertices. The figures indicate that the expected value of density and CC of subgraphs approximate that of the original networks. As expected, the higher coverage, the smaller variance of the characteristics.

References

[1] Salathé, M. *et al.* A high-resolution human contact network for infectious disease transmission. *Proceedings of the National Academy of Sciences of the United States of America* **107**, 22020–22025 (2010).

[2] Albert, R. & Barabási, A.-L. Statistical mechanics of complex networks. *Rev. Mod. Phys.* **74**, 47–97 (2002).

[3] Stumpf, M. P. H., Wiuf, C. & May, R. M. Subnets of scale-free networks are not scale-free: Sampling properties of networks. *Proceedings of the National Academy of Sciences of the United States of America* **102**, 4221–4224 (2005).
